# Surpassing the diffraction limit for improved lateral resolution in adaptive optics optical coherence tomography of the living human eye

**DOI:** 10.1038/s44172-025-00573-5

**Published:** 2025-12-29

**Authors:** Andrew J. Bower, Furu Zhang, Tao Liu, Joanne Li, Nancy Aguilera, Sarah Abouassali, Jonathan Krynitsky, Randy Pursley, Tom Pohida, Bartlomiej Kowalski, Rongwen Lu, Alfredo Dubra, Johnny Tam

**Affiliations:** 1 National Eye Institute, National Institutes of Health, Bethesda, MD USA; 2 National Institute of Biomedical Imaging and Bioengineering, National Institutes of Health, Bethesda, MD USA; 3 Department of Ophthalmology, Stanford University, Palo Alto, CA USA

**Keywords:** Optical imaging, Optics and photonics, Medical imaging, Biomedical engineering

## Abstract

Advances in adaptive optics optical coherence tomography (AOOCT) have facilitated the three-dimensional assessment of structural and functional properties of individual retinal cells in the living human eye. However, even with diffraction-limited AOOCT systems, some cells in the living human retina can be difficult to resolve, especially when using near-infrared wavelengths of light (~1000 nm). We demonstrate that modifying the traditional AOOCT instrument design to enable annular illumination and sub-Airy disk detection results in improved imaging resolution beyond fundamental limits imposed by diffraction. We successfully applied this approach to in vivo human retinal imaging, achieving on average 36% improvement in lateral resolution beyond conventional imaging conditions, enabling improved visualization of the foveal cone and rod photoreceptor mosaics using AOOCT. These results demonstrate an effective strategy for improving lateral resolution in point-scanning AOOCT in a manner that is compatible with new and existing instruments.

## Abstract (web summary)

Bower and colleagues demonstrate sub-diffraction 3D imaging to visualize rods and foveal cones in the living human eye. Their modular strategy can be readily applied to most existing high-resolution ophthalmic imaging systems to improve resolution.

## Introduction

The development of confocal imaging techniques has led to substantial improvements in transverse and axial resolution. By restricting the sample illumination to a small diffraction-limited spot and by spatially filtering the detected light returning from the sample using a confocal pinhole, improved contrast and resolution are achieved^1^. With advances in super-resolution optical microscopy, techniques based on selective modification of illumination^2,3^ and detection^4^ light paths have been demonstrated to visualize structures beyond the diffraction limit according to the Rayleigh criterion.

In confocal retinal imaging of the intact living human eye, resolution is limited by both diffraction as well as by the eye’s imperfect optics (aberrations), which vary not only across eyes^5,6^, but also over time^7^ due to factors such as tear film breakup^8^, eye movement/rotation, and accommodation of the crystalline lens^9,10^. Optical aberrations can be overcome using adaptive optics (AO), which allows for the measurement and correction of aberrations to achieve lateral resolution on the order of 2–3 µm for near-infrared wavelengths^11^. When implemented in a two-dimensional (2D) or three-dimensional (3D) point-scanning confocal imaging configuration (i.e., AO scanning light ophthalmoscopy^12,13^ (AOSLO) and AO optical coherence tomography^14–16^ (AOOCT), respectively), the diffraction-limited imaging performance enables visualization of cellular structures and processes^17–23^ as well as clinical assessment of cellular alterations associated with retinal disease^24–30^. Resolution improvement beyond the diffraction limit has further been demonstrated for AOSLO through modification of illumination^31^, detection^32–36^, or both illumination and detection^37^ light pathways based on annular illumination and sub-Airy disk confocal detection, strategies originally developed for confocal optical microscopy^2,4^. In this paper, we explore the possibility of applying such approaches for point-scanning AOOCT despite differences in image formation processes and instrumentation design.

As many cellular structures lie near the achievable diffraction limit of AOOCT instruments, enhanced lateral resolution may provide additional clarity and improved 3D visualization of retinal cells. However, most lateral resolution enhancement strategies are not easily compatible with most current point-scanning AOOCT designs based on the traditional configuration of the sample arm beam path. In traditional designs, based on a Michelson interferometer, sample arm light delivered to and backscattered from the eye traverse the exact same path in opposite directions before combination with the reference beam, which makes independent modification of the illumination or detection light distributions difficult. Thus, the lack of independent control of illumination and detected light is a limiting factor in implementing resolution-enhancement strategies for AOOCT. To address this, we demonstrate, to our knowledge, the first implementation of sub-diffraction AOOCT imaging in the living human eye based on a Mach-Zehnder architecture to enable independent control of illumination and detection light paths.

## Results

### Decoupling illumination and detection enables resolution enhancement

**Fig. 1:**
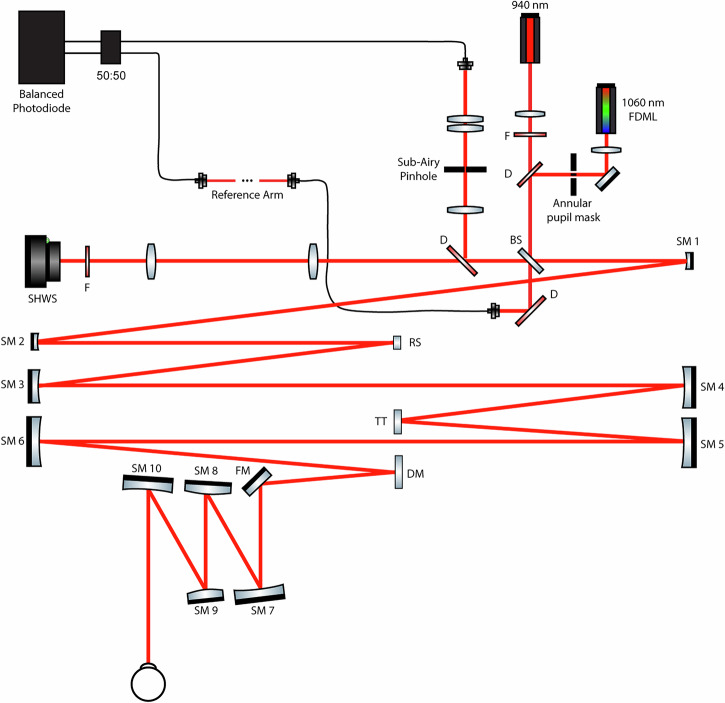
Simplified schematic of the adaptive optics optical coherence tomography (AOOCT) instrument used in this study. Illumination for swept-source OCT is provided by a 3.3 MHz 1060 nm Fourier domain mode-locked laser (FDML) which is combined with the wavefront sensor (WFS) beacon at 940 nm. Illumination and detection light are separated with a 90/10 (transmission/reflection) beamsplitter (BS). 90% of the light returning from the retina is transmitted by the BS and collected in an independent detection arm using a short-pass dichroic mirror (D). Scattered light collected in this detection pathway is interfered with reference arm light for OCT image formation. This approach enables the independent insertion of an annular pupil mask and sub-Airy pinhole into the illumination and detection paths, respectively, to enhance lateral resolution. The optical system is folded in three dimensions based on an “out of the plane” telescope design deployed on a 4.5’ × 7.5’ optical table. Abbreviations: BS beamsplitter, D dichroic mirror, DM deformable mirror, F cleanup filter, FM fold mirror, RS resonant scanner, SHWS Shack-Hartmann wavefront sensor, SM spherical mirror, TT tip/tilt scanner.

We successfully obtained AOOCT volumes after decoupling the illumination and detection paths, using a custom-built AOOCT instrument (see “Methods”; Fig. 1). This approach with decoupled illumination and detection is based on the Mach-Zehnder interferometer architecture, as opposed to the more traditional Michelson interferometer architecture, which features shared illumination and detection paths (Supplementary Table 1; Supplementary Fig. 1).

**Fig. 2:**
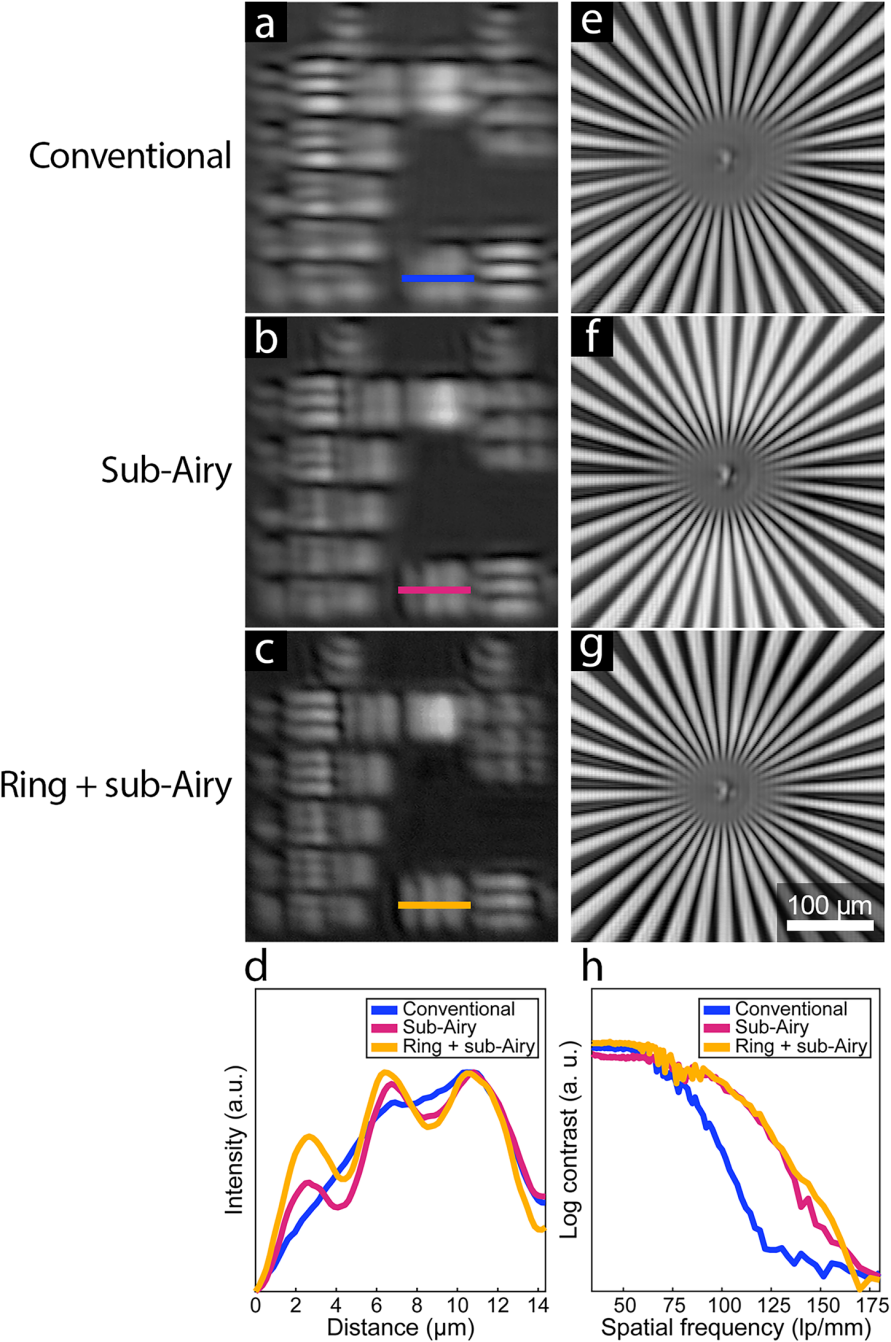
Empirical validation of adaptive optics optical coherence tomography (AOOCT) shows lateral resolution enhancement using 1951 USAF and Siemens star resolution test targets. AOOCT volumes were collected under (**a**, **e**) conventional, (**b**, **f**) sub-Airy, and (**c**, **g**) ring + sub-Airy conditions for each test target. In 1951 USAF target images, improved resolution is observed in the sub-Airy and ring + sub-Airy conditions through increased contrast of bar patterns in group 8. These results are corroborated in images acquired from the Siemens star target where improved resolution is observed under sub-Airy and ring + sub-Airy conditions visualized as enhanced contrast between adjacent bright and dark spokes as the distance between spokes decreases towards the center of the target. For both targets, the best resolution performance was obtained with the ring + sub-Airy condition. Blurring in (**a**–**c**) is due to imaging portions of this target near or beyond the diffraction-limited resolution capabilities of the AOOCT instrument. **d** Line profiles from group 8, element 1 of the 1951 USAF target corroborate qualitative observations demonstrating that the highest contrast is obtained with annular illumination and sub-Airy detection. **h** Contrast between adjacent spokes of the Siemens star target measured across a wide range of spatial frequencies further reveals superior resolution performance is achieved with the sub-Airy and ring + sub-Airy conditions relative to the conventional condition, especially at spatial frequencies greater than 75 lp/mm. Line profiles in (**d**) normalized for visualization by linearly rescaling data from each condition to a common range (0–1).

The decoupled illumination and detection scheme enabled insertion of a sub-Airy (0.7 Airy disk diameter; ADD) pinhole in the detected light pathway without modifying the illumination profile. Consistent with AOSLO and confocal microscopy findings^32^, when the sub-Airy pinhole was inserted in the detection pathway, we observed increased contrast from bar patterns in AOOCT images acquired from a 1951 USAF resolution target (Fig. 2b) compared to the conventional full pupil condition (Supplementary Table 2; Fig. 2a). Likewise, this approach also enabled insertion of a ring-shaped mask to modify the illumination profile, building upon the results demonstrated for confocal microscopy^2^ and AOSLO^31^, with the additional advantage for AOOCT that the light backscattered from the retina is not further attenuated for AOOCT signal detection. When sub-Airy detection was combined with annular pupil illumination (Fig. 2c), resolution was further improved (Fig. 2d), in agreement with and expanding upon previous results in an AOSLO system^37^.

These results were further corroborated through quantitative assessment of images acquired from a Siemens star resolution test target (Fig. 2e–g) which allows optical resolution to be assessed across a wide range of spatial frequencies. Assessment of contrast between adjacent bright and dark spokes with increasing spatial frequency revealed improved contrast at higher frequencies under sub-Airy and ring + sub-Airy conditions corresponding to a 33% increase in spatial frequency cutoff for these conditions relative to the conventional condition (Fig. 2h). Enabled by decoupling the illumination and detection pathways, resolution test target images acquired under sub-Airy and ring + sub-Airy conditions consistently demonstrated enhanced resolution with the best performance observed under the ring + sub-Airy condition, highlighting the value of using a decoupled approach to achieving improved resolution in the living human eye.

### Sub-diffraction imaging of foveal cone photoreceptors in the living human eye

**Fig. 3:**
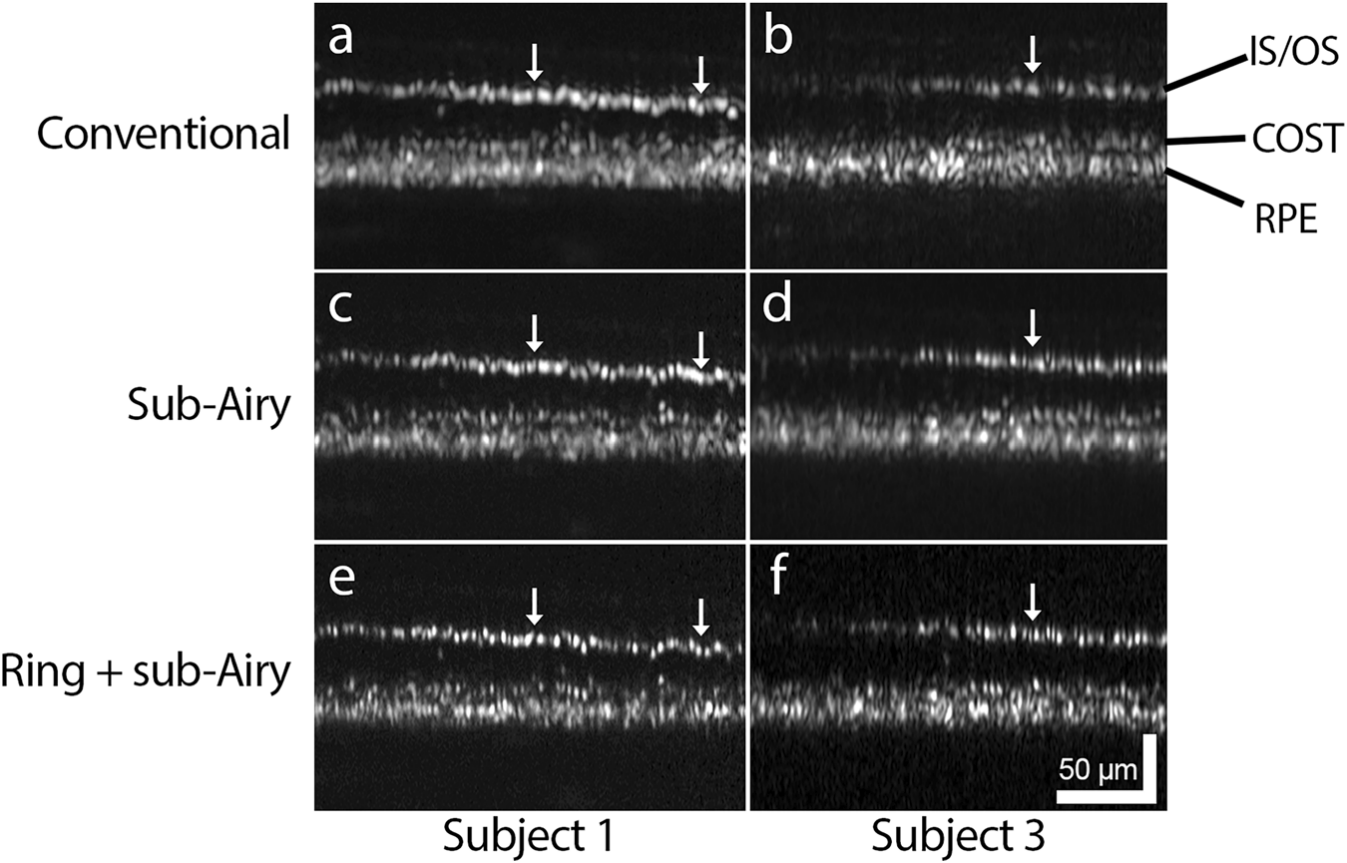
Lateral resolution improvement visualized in adaptive optics optical coherence tomography (AOOCT) B-scans of foveal cones in the living human eye. Averaged B-scans acquired from the fovea of two healthy human subjects co-registered across **a**, **b** conventional, **c**, **d** sub-Airy, and **e**, **f** ring + sub-Airy conditions provide improved visualization of foveal cones. **a**, **b** In conventional images, cone reflections originating from the inner segment/outer segment junction (IS/OS) appear smeared and corrupted by speckle noise (white arrows in (**a**, **b**)). **c**, **d** In images acquired with the sub-Airy pinhole, the smeared appearance in these regions begins to dissipate to begin revealing an array of closely spaced reflections corresponding to foveal cones (white arrows in (**c**, **d**)). **e**, **f** Combination of the sub-Airy pinhole with an annular pupil illumination pattern enables enhanced visualization of these foveal cones (white arrows in (**e**, **f**)) where individual cone photoreceptors can be resolved and distinguished from neighboring cells based on distinct punctate reflections in the IS/OS layer. Weaker reflections are observed from cones very near the foveal center at the left side of each B-scan. Abbreviations: IS/OS inner segment/outer segment junction, COST cone outer segment tips, RPE retinal pigment epithelium.

Having successfully demonstrated improved performance in standard resolution targets, we sought to translate these resolution gains to the living human eye. Depth-resolved AOOCT B-scans were successfully acquired in the living human eye within the fovea, the region of highest cone density (Fig. 3). In conventional B-scans the inner segment/outer segment junction (IS/OS; also referred to as ellipsoid zone band) and cone outer segment tip (COST; also referred to as the interdigitation zone band) bands exhibit a speckled appearance, from which it is difficult to discern features corresponding to individual cones (Fig. 3a, b). Through incorporation of sub-Airy pinhole detection (Fig. 3c, d) and combination with annular pupil illumination (Fig. 3e, f), punctate reflections of individual cone photoreceptors are revealed (Fig. 3, white arrows). The signal-to-noise ratio (SNR) was lower for both sub-Airy (19 dB) and ring + sub-Airy conditions (16 dB) relative to the conventional condition (26 dB) when acquired with equivalent optical power measured at the cornea (limited by light safety considerations as opposed to power loss due to obscuration of the illumination beam; see “Methods”). Nevertheless, even with diminished SNR due to the change in detection scheme (e.g., sub-Airy fiber collection efficiency), it was still possible to successfully visualize cones for all imaging conditions, especially when combined with volume averaging as a common strategy to improve SNR (Supplementary Fig. 2). Thus, eye motion compensation and volume averaging can be used to overcome reduced SNR resulting from annular illumination and/or sub-Airy detection to obtain high-quality sub-diffraction images of the living human retina.

**Fig. 4:**
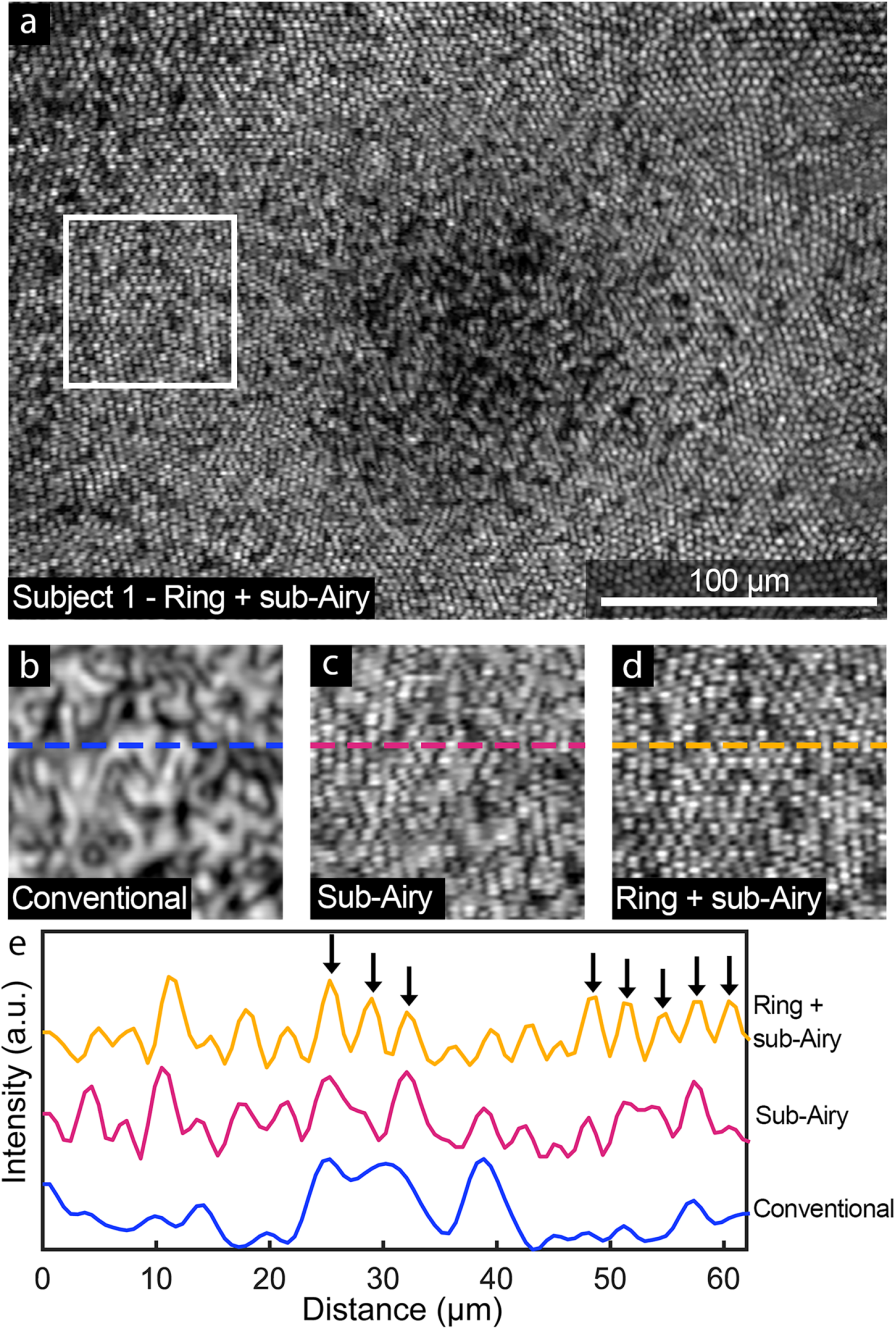
Sub-diffraction adaptive optics optical coherence tomography (AOOCT) imaging reveals the foveal cone mosaic in a healthy subject. **a** En face average volume projection of the inner segment/outer segment junction (IS/OS) layer of Subject 1 obtained from AOOCT volumes acquired with annular pupil illumination and sub-Airy detection. **b**–**d** Side-by-side comparison of average volume projections for the three conditions investigated (region of interest corresponds to the white box in (**a**)) shows a substantial enhancement of the resolution capabilities with both **c** sub-Airy and **d** ring + sub-Airy conditions relative to (**b**) the conventional condition. Interestingly, the appearance of the IS/OS layer in the conventional condition appears as a speckle pattern that is resolved into a tightly packed mosaic of contiguous cones when resolution-enhancing modules are employed. **e** Line profiles corresponding to the dashed lines in (**b**–**d**). Agreeing with qualitative observations, line profiles show the appearance of well-delineated peaks corresponding to foveal cones (black arrows) observed in the ring + sub-Airy condition that are either nearly entirely missing in the case of the conventional condition, or not well-resolved in the sub-Airy condition.

B-scan observations were further corroborated in a densely sampled 3D AOOCT volume acquired from Subject 1 at the fovea (Fig. 4), appearing in en face AOOCT images as a dimmer circular area. En face projection images of the IS/OS layer acquired under the combined annular pupil illumination and sub-Airy detection condition revealed a densely packed contiguous mosaic of cone photoreceptors that can be resolved as close as 60 µm (0.2 degrees) to the foveal center (Fig. 4a). Comparison of a small region of interest co-registered across the three conditions reveals improved resolution performance, demonstrated through improved visualization and clarity of the locally hexagonal mosaic of densely packed foveal cone photoreceptors revealed with the addition of the sub-Airy pinhole (Fig. 4c) and its combination with annular pupil illumination (Fig. 4d) compared to the conventional condition (Fig. 4b). As anticipated from B-scan images, the appearance of the conventional image resembles a speckle pattern, a well-established inherent noise mechanism in OCT due to the interferometric detection scheme^38^. The appearance of this speckle pattern is observed under conventional imaging conditions in areas where resolution performance is insufficient to visualize individual photoreceptors, further obscuring the foveal cone mosaic. As lateral resolution improvement leads to a decrease in the average speckle size^39^, the combined effect of improved lateral resolution and diminished speckle leads to overall improved visualization of foveal cones under sub-Airy and ring + sub-Airy conditions compared to the conventional condition. An example of this is shown in Fig. 4e, where a 1D line profile (measured from the dashed lines in Fig. 4b–d) shows several instances where individual cone photoreceptor reflections are clearly observed in the combined ring + sub-Airy condition but cannot be clearly resolved under conventional or sub-Airy conditions (Fig. 4e black arrows). Qualitatively, cones closest to the fovea were most clearly visualized when combining ring illumination with sub-Airy detection, consistent with resolution targets results (Fig. 2) as well as previous observations with AOSLO^37^.

**Fig. 5:**
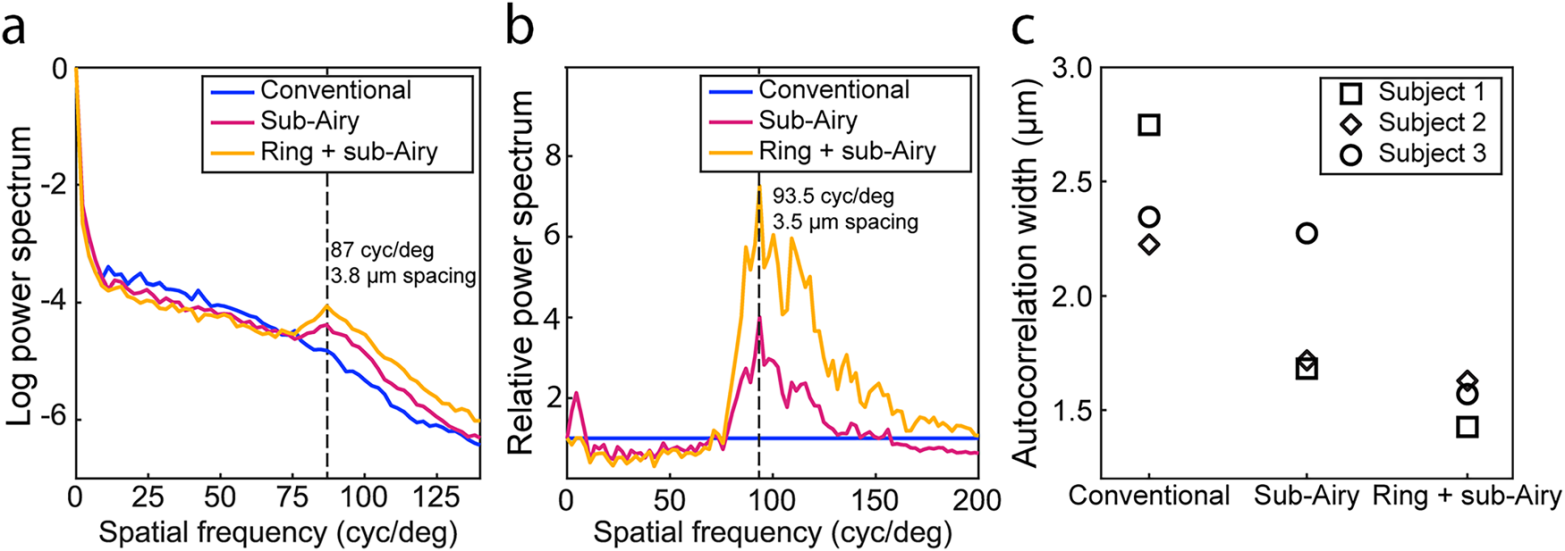
Power spectra and autocorrelation analysis of foveal cones imaged with adaptive optics optical coherence tomography (AOOCT) shows quantitatively improved lateral resolution under sub-Airy and ring + sub-Airy conditions. **a** Radially averaged power spectra obtained from co-registered AOOCT en face projections of the inner segment/outer segment junction (IS/OS) layer acquired under each imaging condition in Subject 3. While not observed in the conventional condition, a prominent peak in the power spectra of the sub-Airy and ring + sub-Airy conditions are observed corresponding to a cell-to-cell spacing of 3.8 µm. **b** Closer examination of the relative power spectra reveals a substantial improvement in resolution performance for both the sub-Airy and ring + sub-Airy conditions with the latter exhibiting the greatest improvement relative to the conventional condition corresponding to a cell-to-cell spacing of 3.5 µm. **c** Autocorrelation width (full width at half maximum) of AOOCT en face foveal cone images obtained for all subjects and conditions. For each subject, the calculated autocorrelation width decreases with the incorporation of resolution-enhancing modules, exhibiting the smallest value under the combined ring + sub-Airy condition, indicating the superior resolution performance obtained under this condition.

To quantitatively assess the resolution performance, radial power spectra of foveal cone mosaics were successfully generated and compared. While no clear peak corresponding to the cell spacing of foveal cone mosaic can be clearly observed in the conventional power spectrum, prominent peaks can be clearly discerned under the sub-Airy and ring + sub-Airy conditions with the combined approach showing the most prominent peak corresponding to a cell spacing of 3.8 µm (Fig. 5a). Additionally, close inspection of the relative power spectra (in reference to the conventional condition—see “Methods” for definition) reveals a 7x increase at its peak, corresponding to a cell spacing of 3.5 µm (Fig. 5b). Analysis of the autocorrelation width of each condition (Fig. 5c) further corroborates these findings, demonstrating decreased autocorrelation width, corresponding to improved lateral resolution with the smallest widths corresponding to the ring + sub-Airy condition. Together, these results provide evidence for improved lateral resolution in 3D AOOCT imaging of foveal cones with annular pupil illumination and sub-Airy pinhole detection. Furthermore, these results indicate the potential for improved imaging of other cellular structures whose sizes are near the resolution limit imposed by the living human eye.

### Enhanced visualization of rod photoreceptors

**Fig. 6:**
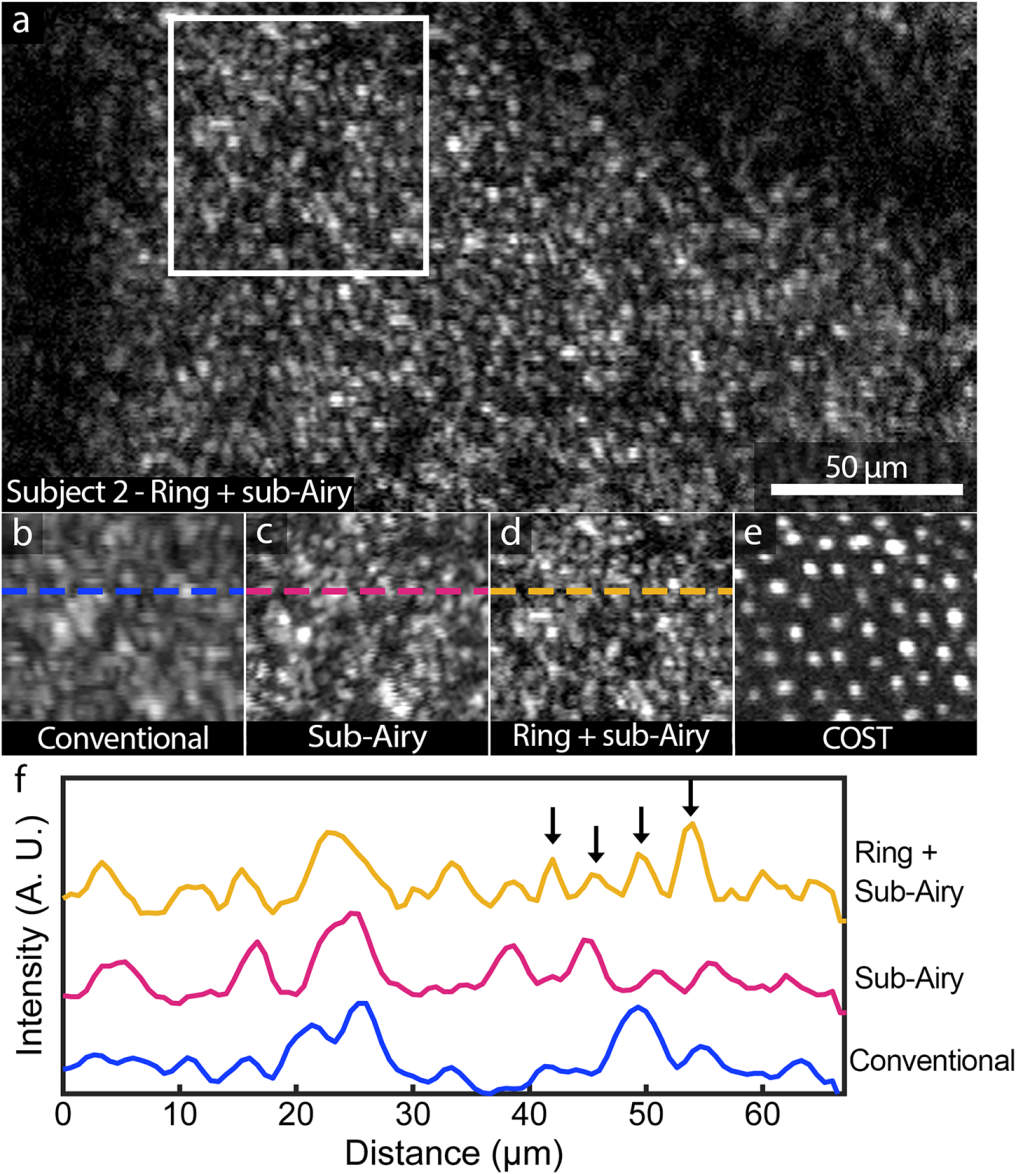
Annular illumination and sub-Airy pinhole detection enhances resolution in adaptive optics optical coherence tomography (AOOCT) images of the rod mosaic. **a** En face projection of the rod outer segment tip (ROST) layer obtained from AOOCT volumes acquired with annular pupil illumination and sub-Airy detection (Subject 2). **b**–**d** Side-by-side comparison of the three conditions (corresponding to the region denoted by the white box in (**a**) shows enhancement of the resolution capabilities achieved using both **c** sub-Airy and **d** ring + sub-Airy conditions relative to (**b**) the conventional condition. Similar to observations from foveal cones, conventional images show a smeared reflectance pattern corrupted by speckle. With the addition of annular illumination and sub-Airy detection, individual punctate reflections can be distinguished corresponding to rods, surrounded by larger darker circular areas corresponding to the overlying cone photoreceptor mosaic. **e** En face projection of the cone outer segment tips (COST) layer for comparison. **f** Line profiles obtained corresponding to the dashed lines in (**b**–**d**). In agreement with qualitative observations and similar to foveal cone observations, line profiles show the appearance of well-resolved peaks corresponding to rod photoreceptors observed in the ring + sub-Airy condition that are not clearly delineated in either of the other conditions.

**Fig. 7:**
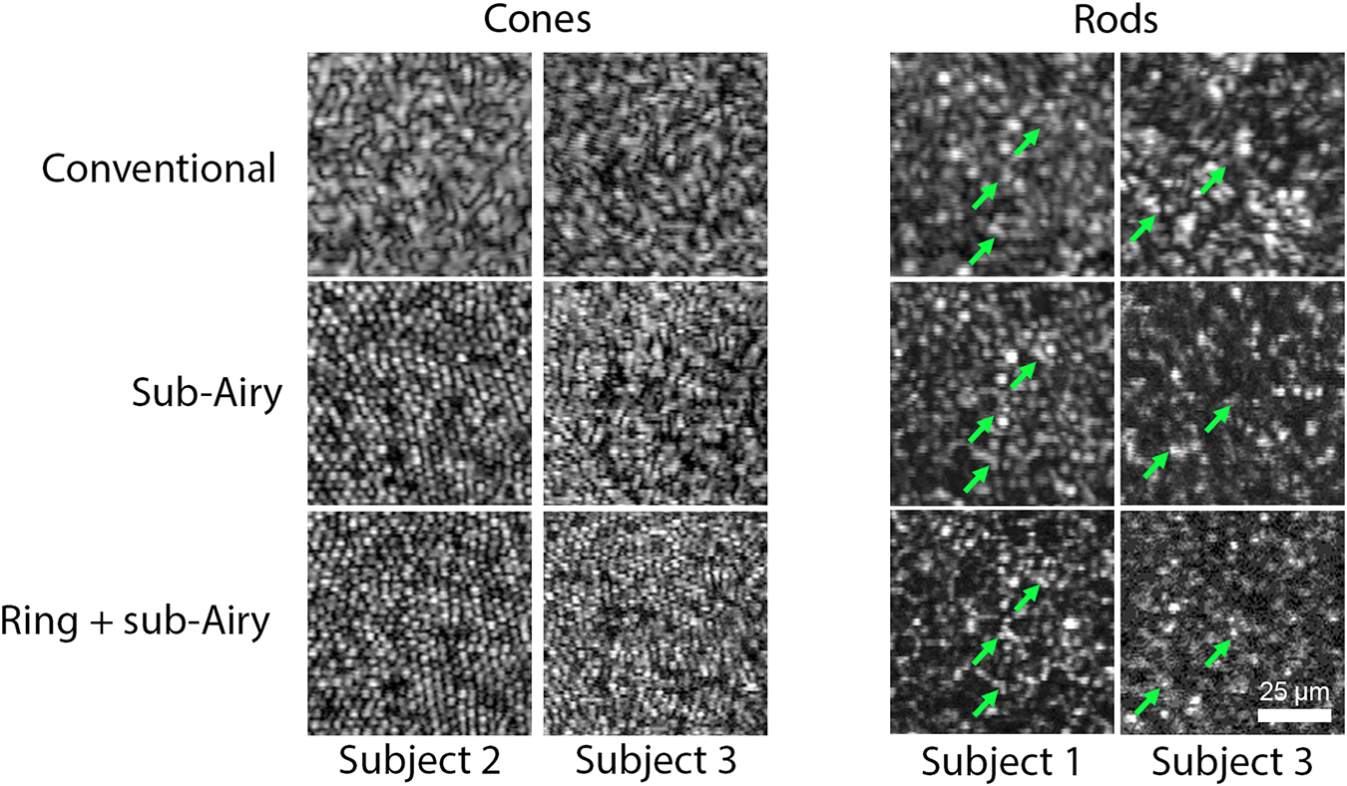
Additional examples of cone and rod photoreceptor images acquired for each subject. (Left panel) Images acquired within 0.5° of the foveal center of subjects 2 and 3 reveal a hexagonal mosaic of cone photoreceptors under sub-Airy and ring + sub-Airy conditions compared to the conventional condition where the cone mosaics are obscured by speckle. (Right panel) Images acquired at the rod outer segment tips (ROST) layer in subjects 1 and 3 at retinal eccentricities of 1.5 and 2.0 mm temporal to the fovea, respectively. Rod photoreceptors are observed as punctate reflections surrounding dark circular areas corresponding to overlying cone photoreceptors visible under sub-Airy and ring + sub-Airy conditions but obscured under the conventional condition. Green arrows denote specific examples where improved rod photoreceptor visualization is observed under resolution-enhanced conditions. For all subjects and retinal locations assessed, the best lateral resolution is observed with the combined use of annular pupil illumination and sub-Airy detection.

Along with the improved resolution of foveal cone photoreceptors, improved visualization of rod photoreceptors was observed in human subjects at retinal eccentricities 1.5–2.0 mm temporal to the fovea. The high axial resolution of AOOCT, which permits depth-resolved imaging, enables the backscattered signal from rod outer segment tips (ROST) to be separately isolated from reflections of other retinal layers, including the overlying cone mosaic (Supplementary Fig. 3). Examination of the en face projection of the ROST layer acquired with combined annular pupil illumination and sub-Airy pinhole detection (Fig. 6a, Supplementary Fig. 3b) reveals a mosaic of closely spaced cells surrounding dark circular structures corresponding to overlying cone photoreceptors (Fig. 6e, Supplementary Fig. 3a). As observed in foveal cone images, the best resolution performance is observed under the ring + sub-Airy condition (Fig. 6b–d). Interestingly, conventional images of the rod mosaic (Fig. 6b) also exhibit a characteristic speckle appearance obscuring the appearance of both individual rods and the dark circular areas corresponding to cone photoreceptors. Line profiles obtained from the ROST layer show several instances where individual rods are well delineated under combined annular pupil illumination and sub-Airy detection but cannot be distinguished under the conventional condition or sub-Airy detection alone (Fig. 6f). We further corroborated results of improved photoreceptor imaging in additional subjects (Fig. 7) where sub-Airy and ring + sub-airy conditions provided enhanced lateral resolution with the best lateral resolution performance observed under the ring + sub-Airy condition for all subjects for both cone and rod photoreceptor imaging.

**Fig. 8:**
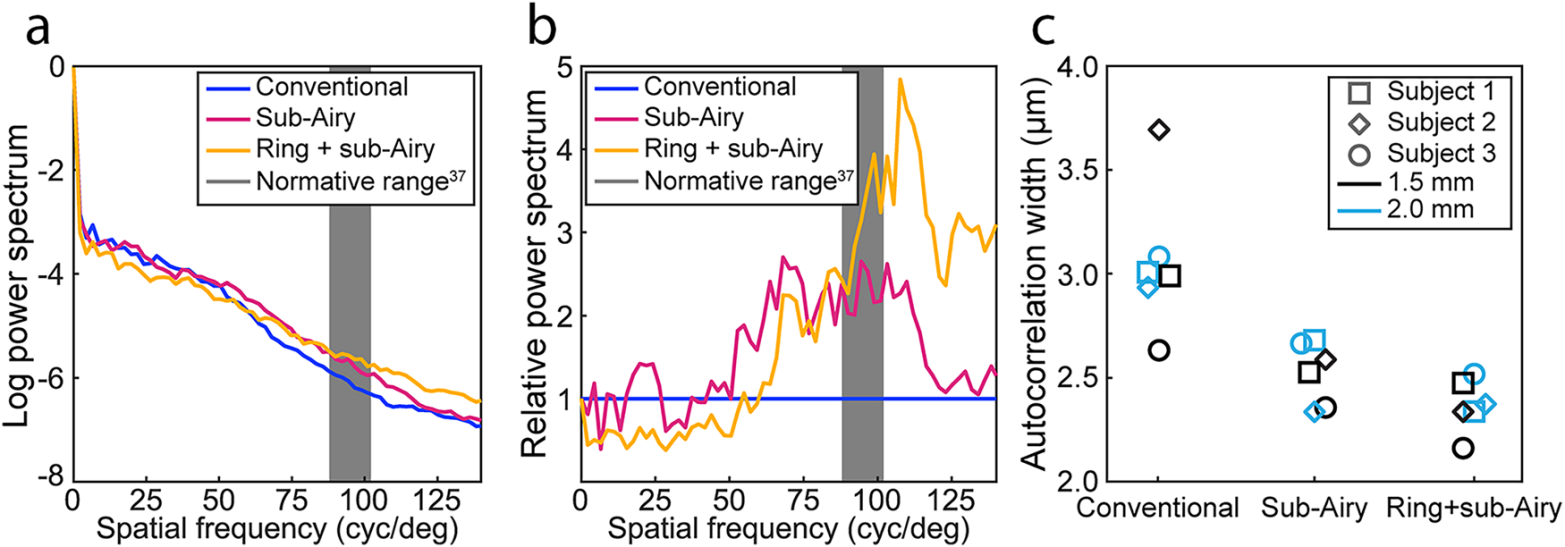
Power spectra and autocorrelation analysis of rod photoreceptors show improvement in lateral resolution with incorporation of annular illumination and sub-Airy pinhole detection. **a** Radially averaged power spectra obtained from co-registered adaptive optics optical coherence tomography (AOOCT) en face projections of the rod outer segment tips (ROST) layer acquired under each imaging condition 2.0 mm temporal to the fovea of Subject 1. No peak corresponding to rod spacing could be clearly observed in the power spectra acquired under any of the imaging conditions. **b** Relative power spectra calculated for each condition relative to the conventional condition. For spatial frequencies greater than ~50 cycles/degree, power spectra obtained from images acquired under sub-Airy and ring + sub-Airy conditions were consistently greater than those obtained under the conventional condition. **c** Autocorrelation width (full width at half maximum) of AOOCT en face rod photoreceptor images acquired from each subject at 1.5 mm and 2.0 mm temporal to the fovea for each imaging condition. With the incorporation of resolution-enhancing modules, the calculated autocorrelation width is decreased reaching its minimal value obtained under the combined ring + sub-Airy condition. Gray shaded regions in (**a**, **b**) correspond to normative rod density data^63^.

In contrast to foveal cone assessment, no discernable peaks were observed in power spectra corresponding to rod spacing under any of the conditions investigated (Fig. 8a). As with power spectra obtained from foveal cone mosaics, the sub-Airy and ring + sub-Airy conditions showed improved resolution relative to the conventional condition at higher spatial frequencies corresponding to smaller cell spacings (Fig. 8b) with the ring + sub-Airy condition exhibiting the greatest relative increase at higher spatial frequencies. Corroborating these results, we also observed similar trends in the autocorrelation width (full width at half maximum) of rod mosaics (Fig. 8c) as was observed in foveal cone mosaics with the smallest autocorrelation widths (best resolution performance) observed for the ring + sub-Airy condition. Together, these results corroborate observations made from foveal cone imaging and further demonstrate the extension of lateral resolution improvement to other cell layers of the living human retina.

## Discussion

In this study, we reexamine the traditional AOOCT optical design and demonstrate that a Mach-Zehnder architecture may have some benefits over the traditional Michelson interferometer^20,22,40–45^ used for point-scanning AOOCT (Supplementary Fig. 1). To our knowledge, this is the first demonstration of decoupling and independently modifying illumination and detection pathways in OCT to enhance lateral image resolution. While the optical design of our modified point-scanning instrument is based on the underlying principle of confocal imaging, distinct image formation processes in AOOCT lead to unique features in the acquired data. AOOCT, based on combining broadband illumination with interferometric detection of backscattered light, generates a depth-resolved 3D volumetric dataset permitting visualization of the laminar structure of the human retina with high axial resolution. Despite these fundamental differences in image formation, we were able to successfully achieve sub-diffraction limit imaging in AOOCT using annular illumination combined with sub-Airy detection.

The use of optical resolution enhancement strategies to enable imaging of foveal cone and rod photoreceptors has been the focus of many studies using AOSLO^31,32,37,46,47^. There have not, however, been any studies demonstrating optical strategies to enhance AOOCT lateral resolution to our knowledge. Beyond the 2D imaging capabilities of AOSLO, 3D imaging of foveal cone and rod photoreceptor mosaics can be enabled by AOOCT if sufficient lateral resolution can be achieved. By using this modified AOOCT design to independently illuminate the subject’s pupil with an annular illumination profile and restricting the spatial extent of light returning from the retina with a sub-Airy confocal pinhole, we achieved lateral resolution enhancement beyond the diffraction limit permitting improved 3D visualization of cells that are near the fovea in the living human retina. While both the sub-Airy pinhole and annular illumination strategies can enable improved lateral resolution, the use of annular illumination alone may distort the appearance of cone photoreceptors (Supplementary Fig. 4b) due to the increased relative intensity present in the sidelobes of the point spread function. Therefore, if only one of these elements is to be implemented, the sub-Airy pinhole is preferrable to avoid imaging artifacts. Across all experiments, however, we consistently achieved the best resolution performance when combining annular pupil illumination with sub-Airy pinhole detection, which showed substantial improvement over both the conventional condition and the sub-Airy detection condition alone. As closed-loop AO performance was similar across all imaging conditions (Supplementary Fig. 4), the improvements to lateral resolution observed in this study can be attributed to the use of annular illumination and/or sub-Airy pinhole modules. With further refinement, this approach may enable clinical assessment of foveal cone and rod photoreceptors in the living human eye even at higher wavelengths, such as for precise visualization of 3D cellular structures in disease^43,48^.

The numerical aperture of the human eye varies from subject to subject, affected by biometric parameters such as axial length and dilated pupil diameter. Thus the achievable diffraction limit will also vary from subject to subject^49^, but is expected to be ~2–3 µm for systems operating at central wavelengths of 800–1100 nm^37^. Based on the pupil size used in this study (7.7 mm), and assuming an equivalent focal length of 16.667 mm from the Bennett-Rabbetts simplified model eye^50^, we estimate the theoretical lateral resolution of the AOOCT instrument used in this study to be 2.8 µm in the human eye. To achieve this diffraction limit in practice, correction of optical aberrations using AO is needed and serves as an important prerequisite for achieving sub-diffraction limited resolution. Along with the sub-diffraction lateral resolution, a key advantage of OCT imaging is the decoupling of axial and lateral resolution. Specifically, axial resolution only depends on the spectral properties of the light source, while lateral resolution, assuming successful AO correction of any present ocular aberrations, is dependent on the numerical aperture of the subject’s eye, the illumination light distribution, and the detection pinhole size. Therefore, we can improve lateral resolution by employing these imaging modules without compromising the inherent high axial resolution of AOOCT.

An important consequence of the interferometric detection scheme used to obtain this high axial resolution is the generation of speckle noise, a fundamental property of all OCT systems that is not commonly observed in modern AOSLO instruments. In general, speckle may be observed in imaging systems as interference between multiple coherent scattered waves^38^. In AOOCT, speckle is primarily due to detection of multiply scattered coherent light and was clearly observed in this study under conventional AO imaging conditions when imaging foveal cone (Fig. 4B and Supplementary Movie 1) and rod (Fig. 6b) photoreceptors. In these images, cellular structures take on an unpredictable smeared appearance. Our results show that insertion of resolution-enhancing elements has a secondary benefit in further reducing speckle in AOOCT. For all subjects, the effects of speckle noise (smeared appearance of cellular structures) were least prevalent under the ring + sub-Airy condition. Thus, annular illumination and/or sub-Airy detection not only improves lateral resolution but also reduces the effects of speckle noise, enabling improved visualization of more cells in the living human eye. Corroborating these in vivo observations, previous work has compared AOSLO and AOOCT of structures lying near the optical resolution limit using 3D printed phantoms and found similar speckle patterns dominating AOOCT images^51^. As may be expected, this speckle pattern was not observed in corresponding AOSLO phantom images. This suggests that the use of resolution-enhancing elements may present greater advantages in AOOCT in visualizing structures lying near the optical resolution limit by simultaneously improving lateral resolution and decreasing the effects of speckle.

To achieve further lateral resolution gains, the strategies used here to enable improved resolution may be extended to and combined with other approaches. For example, a more restrictive (smaller diameter) sub-Airy pinhole and a narrower annular illumination profile would in theory provide improved lateral resolution. However, the resolution benefits of smaller sub-Airy pinholes and narrower annular illumination profiles will be accompanied by corresponding decreases in SNR (Supplementary Fig. 2), which may be especially challenging for imaging lower contrast cellular structures in the retina^32^. Our choice of 0.7 ADD was informed by our previous AOSLO study which found this pinhole size balanced both SNR and resolution considerations in imaging the living human retina^37^. While averaging can be used to boost SNR, even small amounts of eye motion can shift the image area resulting in blurring, which has the effect of reducing apparent lateral resolution. To overcome this challenge, we use a strip-based registration approach here to precisely co-register individual AOOCT volumes to a template volume using a strip-based normalized cross-correlation alignment. If the SNR of a single volume is sufficient to permit precise strip placement, as is the case in all imaging conditions shown here, averaging of registered AOOCT volumes can be used to boost SNR without compromising lateral resolution.

The 1060 nm swept wavelength light source used here was chosen for practical considerations to improve the overall imaging speed of the system (3.3 MHz A-scan rate) in a point-scanning design, faster than existing point-scanning spectral-domain approaches that typically image near 800 nm (limited by spectral domain detector speeds). Although the increased speed for swept source systems typically comes at the expense of lower lateral resolution (~30% based on the different wavelengths), by implementing the annular illumination and sub-Airy confocal detection strategy, we demonstrate that it is possible to simultaneously obtain the increased speed associated with using a swept source system alongside resolution that is comparable to that which would have been achieved using spectral domain OCT. However, it is important to note that faster acquisition speeds and shorter central wavelengths may be possible in both AOSLO and AOOCT to improve imaging performance. By implementing the architecture and resolution-enhancing modules demonstrated here, other existing instruments based on shorter central wavelengths, for example near 800 nm^44,52^, may enable even finer resolution performance to visualize even smaller cells as well as subcellular structures of the living human eye. Beyond applications in the eye, this resolution enhancement strategy is generalizable to any point-scanning imaging based on confocal detection including optical coherence microscopy^53^, for assessing sub-resolution features in tissues such as brain^54^, skin^55^, or embryonic tissues^56^.

While point-scanning AOOCT enables 3D imaging based on a unique image formation process, the optical design of such systems is centered on the fundamental principle of confocal detection. To overcome traditional design limitations, we demonstrate here lateral resolution improvement beyond the optical diffraction limit enabled by modifying the traditional architecture of AOOCT, enabling 3D visualization of more retinal cells in the living human eye.

## Methods

### AOOCT instrumentation

A custom swept-source point-scanning AOOCT instrument (Fig. 1) was designed and constructed based on a Fourier domain mode-locked light source (FDML) with a sweep rate (A-scan rate) of 3.3 MHz, central wavelength of 1060 nm, and bandwidth of 77 nm (NG-FDML-1060-750-8B-FA, Optores, Munich, Germany). The 1060 nm FDML light was combined with a wavefront sensor (WFS) beacon at 940 nm (SLD-mCS-481-HP2-PM, Superlum, Carrigtwohill, Co. Cork, Ireland). The measured optical power at the cornea was maintained below 1.6 mW for the 1060 nm FDML source and below 105 µW for the 940 nm WFS beacon. Used in combination, the optical power of these sources lies below the maximum permissible exposure defined by the American National Standards Institute standard^57^. The coaxially aligned 940 and 1060 nm beams are raster scanned in the horizontal direction using a 3.3 kHz resonant scanner (SC-30, Electro-Optical Products Corp, Fresh Meadows, NY, USA) and in the vertical direction using a tip-tilt scanner (S-334, PI-USA, Auburn, MA, USA). The Shack-Hartmann WFS used in this study is based on a similar design used for in vivo human AO retinal imaging previously^13^. Fundamentally, the dynamic range of the Shack-Hartmann WFS is limited by the overlap of images generated by each individual lenslet^58^. However, the Shack-Hartmann WFS has historically been used to characterize monochromatic aberrations in relatively large cohorts of human subjects^6^ and has been widely used in both AOSLO and AOOCT retinal imaging. Wavefront correction is performed using a deformable mirror (DM97, Alpao, Montbonnot, France), capable of wavefront correction over a nominal range ±8 diopters of defocus at a closed-loop correction rate of up to 8 Hz. The beam size at the subject’s pupil was 7.7 mm. To facilitate the independent incorporation of resolution-enhancing modules, the illumination and detection pathways in the system are decoupled. Light from the illumination pathway is coupled into the optical system through a 90/10 (transmission/reflection) beamsplitter (BS; BSN11, Thorlabs, Newton, NJ, USA). After descanning, backscattered light from the retina is routed to a separate detection pathway and through an interchangeable pinhole, enabling assessment of lateral resolution across different pinhole sizes. Light transmitted by the pinhole is collected in a single-mode optical fiber and sent to a balanced photodiode (PDB481C-AC-SP2, Thorlabs, Newton, NJ, USA) where it is interfered with a reference beam for OCT detection. The optical system, folded in three dimensions based on an “out of the plane” telescope design^13^, is deployed on a 4.5’ × 7.5’ optical table in the NEI eye clinic. To assess lateral resolution improvements, we compared three imaging conditions, described in Supplementary Table 2, throughout the study. Closed-loop AO operation was maintained across all imaging conditions throughout the study.

### Resolution test target imaging

A 1951 USAF resolution test target (#58-198, Edmund Optics, Barrington, NJ, USA) and a Siemens star target (#37-538, Edmund Optics, Barrington, NJ, USA) were used to assess and compare the AOOCT resolution performance under each condition (Supplementary Table 2). Resolution was assessed with the USAF test target by comparing the visibility and contrast of the same closely spaced bar pattern under each imaging condition. The Siemens star target allows optical resolution to be assessed in a more continuous fashion with spatial frequency. In this target, resolution is assessed by analyzing the contrast of adjacent spokes traced using a circular line profile centered on the convergence point of the spokes. As the spatial frequency of the spoke pattern increases towards the center of the target, contrast between adjacent spokes is evaluated continuously with spatial frequency to assess the resolution performance for each condition.

### AOOCT imaging in human subjects

Human subjects were recruited through the National Institutes of Health Clinical Center for AOOCT imaging (Supplementary Table 3). A comprehensive dilated eye examination was performed to confirm that there were no signs of ocular disease. Research procedures adhered to the tenets of the Declaration of Helsinki and were approved by the local Institutional Review Board (National Institutes of Health). Written informed consent was obtained for all participants. Eyes were dilated with 2.5% phenylephrine hydrochloride (Akorn, Lake Forest, IL, USA) and 1.0% tropicamide before imaging (Sandoz, Basel, Switzerland). AOOCT videos were acquired at 12.7 volumes per second with 512 × 512 A-scan sampling across a 1.0 degree square field-of-view. AOOCT volumes were obtained at the foveal center and at eccentricities ranging from 1.5 to 2.0 mm temporal to the fovea for each subject. Ten videos, each consisting of 17 AOOCT volumes, were acquired at each eccentricity for each imaging condition (Supplementary Table 2). For conditions in which the illumination pattern was changed (ring illumination), the optical power of the 1060 nm FDML was adjusted to maintain the equivalent optical power at the cornea as full beam illumination. Sequential imaging with the same protocol was repeated at matching retinal eccentricities for each condition.

### Image processing and analysis

To compensate for axial eye motion, B-scans from each AOOCT volume were digitally flattened based on reflections from the outer retinal layers and axially aligned. To correct for angular tilt of the retinal image, AOOCT B-scan images were thresholded, binarized, and fit with an ellipse to determine the relative tilt angle. Each A-scan was then shifted by a corresponding number of pixels to correct for the calculated tilt angle. Lateral eye motion was compensated using strip-based image registration^59^. In this step, a 2D en face projection image of the photoreceptor layer was generated for each AOOCT volume and used to compensate for lateral eye motion using strip-based image registration^60^, which was then applied to the entire 3D volume before averaging. En face images were extracted for further analysis by maximum intensity projection through retinal layers of interest of the corresponding averaged AOOCT volume. To obtain the retinal scaling factor from angle in degrees to millimeters, a paraxial ray trace on a three-surface eye model^50^ was performed after updating the model parameters with the axial length, corneal curvature, and anterior chamber depth measured for each eye (IOL Master, Carl Zeiss Meditec, Dublin, CA, USA).

The spatial frequency content of photoreceptor images acquired under each imaging condition was compared using the radially averaged 2D power spectrum^61^. For foveal cone images, cell packing metrics were calculated by identifying the spatial frequency corresponding to the most prominent peak in the power spectrum. Cell-to-cell spacing was estimated assuming a hexagonal packing of foveal cones^62^. To provide direct comparisons between the imaging conditions, plots of relative power spectra were constructed by dividing radially averaged power spectra by the power spectrum obtained from images acquired under the conventional condition. The full width at half maximum of the image normalized autocorrelation was used as an additional measure to evaluate resolution performance for each condition as described previously^31,37^. To ensure direct comparisons, images acquired under each condition were manually co-registered and corresponding regions of interest were selected for analysis prior to calculation of the normalized autocorrelation.

### Reporting summary

Further information on research design is available in the Nature Portfolio Reporting Summary linked to this article.

## Supplementary information

Supplemental Material

Description of Additional Supplementary Files

Supplemental Movie 1

Reporting Summary

## Data Availability

All data needed to evaluate the conclusions in the paper are present in the manuscript and/or supporting information.
