# Supplementary material for: Surpassing the diffraction limit for improved lateral resolution in adaptive optics optical coherence tomography of the living human eye
Source: Commun Eng. 2025 Dec 29;5:3. doi: 10.1038/s44172-025-00573-5 (PMC12764984; doi:10.1038/s44172-025-00573-5)
Supplement: Supplementary file 1 — Supplemental Material [file 44172_2025_573_MOESM1_ESM.pdf]

Supplementary Information

Surpassing the diffraction limit for improved lateral resolution in adaptive optics optical coherence tomography of the living human eye

Andrew J. Bower[1], Furu Zhang[1], Tao Liu[1], Joanne Li[1], Nancy Aguilera[1], Sarah Abouassali[1], Jonathan Krynitsky[2], Randy Pursley[2], Tom Pohida[2], Bartlomiej Kowalski[3], Rongwen Lu[1], Alfredo Dubra[3], Johnny Tam[1*]

[1] National Eye Institute, National Institutes of Health, Bethesda, MD 20892, USA.
[2] National Institute of Biomedical Imaging and Bioengineering, National Institutes of Health, Bethesda, MD 20892, USA.
[3] Department of Ophthalmology, Stanford University, Palo Alto, CA 94303, USA.

*Corresponding author. Email: johnny@nih.gov

This PDF file includes:

Supplementary Figures 1 to 4

Supplementary Tables 1 to 3

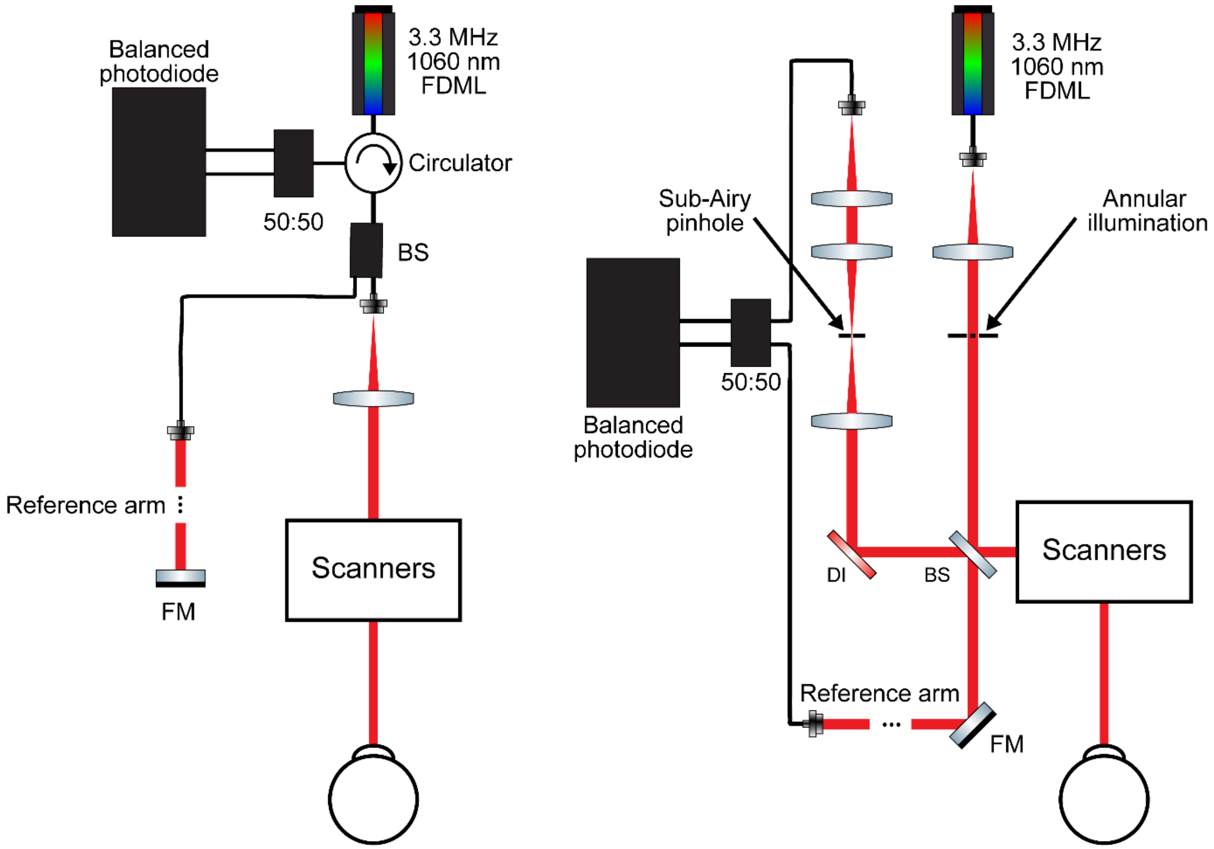

Supplementary Figure 1. Example system schematics comparing (left) shared/traditional (based on a Michaelson interferometer design) and (right) decoupled illumination/detection (based on a Mach-Zehnder interferometer design) for adaptive optics optical coherence tomography (AOOCT) imaging. Signal loss is expected when altering the illumination or detected light distributions in the shared pathway design due to the double-pass configuration. Alternatively, decoupling the illumination and detection pathways enables insertion of optical elements to modulate the illumination and detection light distributions independently. Abbreviations: BS – beamsplitter; DI – dichroic mirror; FDML – Fourier domain mode-locked laser; FM – flat mirror.

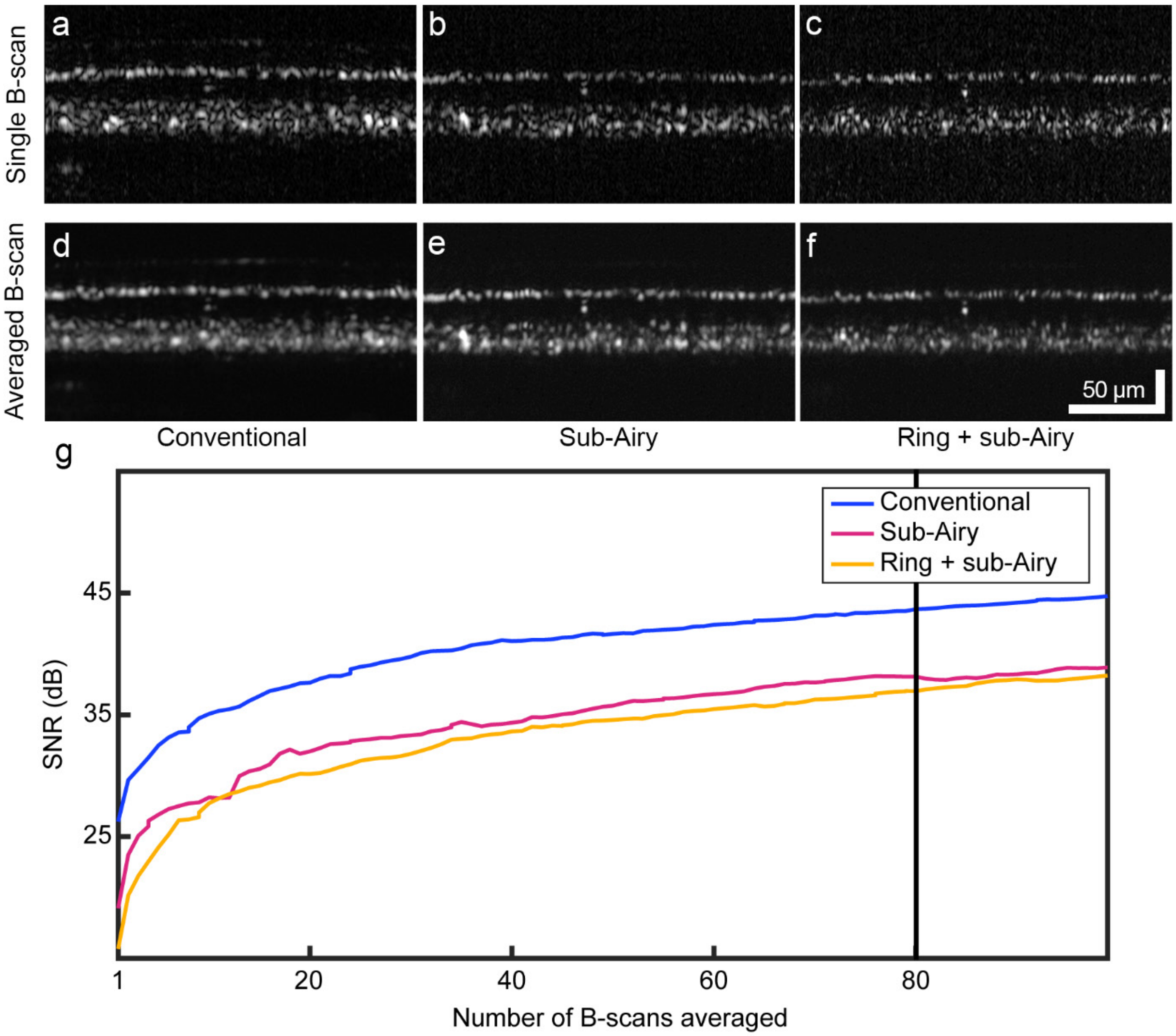

Supplementary Figure 2. **Signal-to-noise ratio (SNR) is sufficient for visualization of cells and improves with volume averaging across all imaging conditions**. (a-c) Single B-scans acquired under (a) conventional, (b) sub-Airy, and (c) ring + sub-Airy conditions (Subject 1). Compared to the conventional condition, images acquired under sub-Airy and ring + sub-Airy conditions exhibit decreased SNR. (d-f) Averaged B-scans (n = 80) acquired under each condition demonstrate improvements in SNR achieved by averaging under (d) conventional, (e) sub-Airy, and (f) ring + sub-Airy conditions. (g) Measurements of SNR improvement with averaging for each imaging condition. While decreased SNR was observed from conditions that provide enhanced lateral resolution, the SNR obtained from a single volume was sufficient to achieve successful strip-registration and averaging to increase SNR. Black vertical line in (g) corresponds to number of averages used for comparison in (d-f). SNR is calculated as $SNR = 20 * log(\mu_{sig}/\sigma_{noise})$ where μ_{sig} is the mean signal and σ_{noise} is the standard deviation of the noise in the region analyzed.

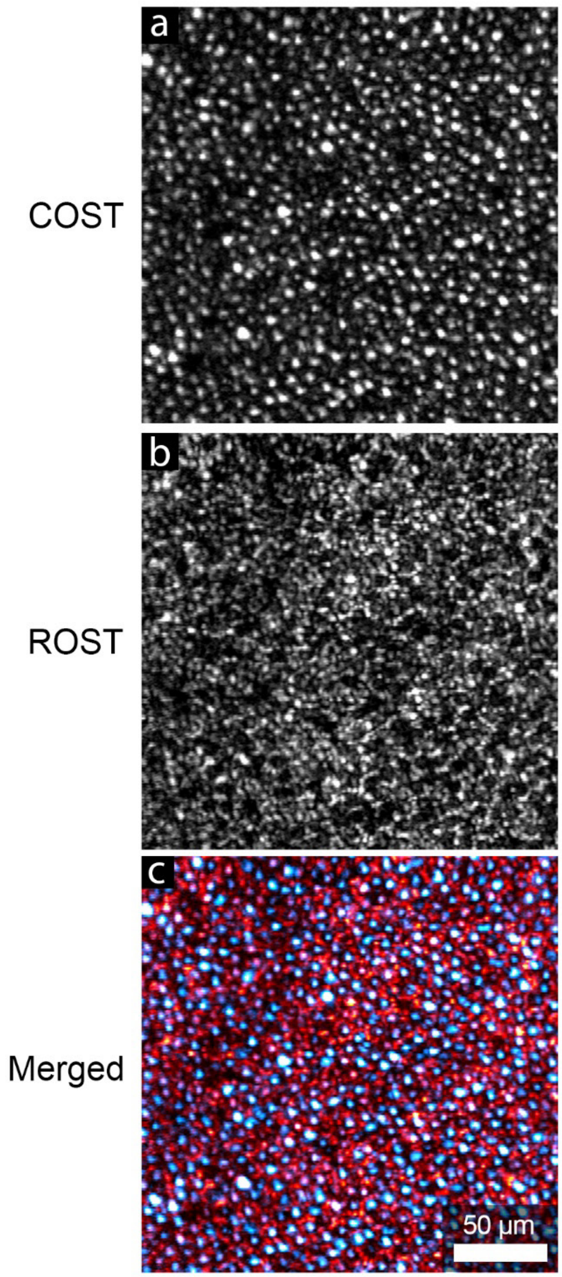

Supplementary Figure 3. Enhanced lateral resolution enables delineation of cone and rod photoreceptors. Using the depth sectioning capabilities of AOOCT, cones and rods can be isolated and clearly delineated by separately creating en face projection images of the (a) cone outer segment tip (COST) and (b) rod outer segment tip (ROST) layers separately (Subject 1). (c) Color-merged composite shows an expected arrangement of photoreceptor cells with a collection of tightly spaced rods (red) surrounding each cone cell (blue).

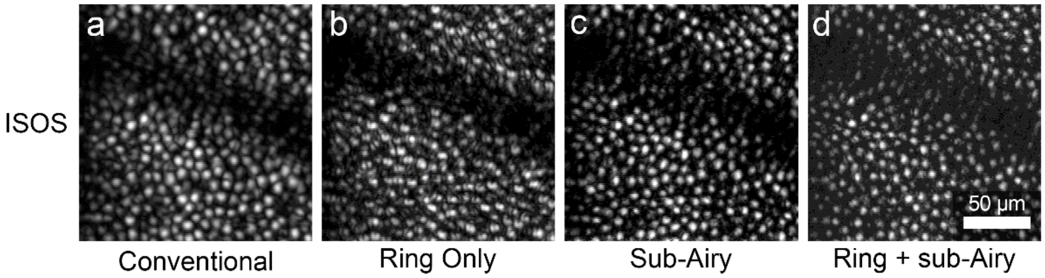

Supplementary Figure 4. Comparison of AOOCT cone photoreceptor images acquired with different combinations of resolution-enhancing elements. Images were acquired at an eccentricity of 1.5 mm (Subject 2) under (a) Conventional, (b) Ring only, (c) Sub-Airy, and (d) Ring + sub-Airy conditions. At this eccentricity, cones are larger relative to diffraction-limited performance enabled by closed-loop AO correction. Visualization of cone photoreceptors in (a-d) demonstrates similarly effective AO performance for each imaging condition. However, sidelobes are apparent in (b) leading to a "halo-like" appearance of cones due to the use of an annular illumination mask. These artifacts are mitigated through (d) combination of annular illumination and sub-Airy pinhole.

Supplementary Table 1. Comparison of shared (traditional) and decoupled (used in this study) illumination/detection designs

**Shared/traditional illumination/detection (Michelson interferometer)**	**Decoupled illumination/detection (Mach-Zehnder interferometer)**
OCT often separated from other light sources and aligned separately	Co-axial alignment of all optical sources prior to coupling into optical system
Optical elements placed in shared pathway affects both illumination and detected light distributions	Optical elements can be independently inserted to alter the illumination or detected light distributions
Reference arm light typically generated using a separate beamsplitter or fiber coupler	Reference arm light naturally separated and collected using the system 90:10 beamsplitter

Supplementary Table 2. Illumination and detection parameters for each imaging condition used in this study

Condition	Illumination profile (ε = inner/outer diameter)	Detection pinhole size (ADD)
Conventional	Circular (ε = 0)	1.2
Sub-Airy	Circular (ε = 0)	0.7
Ring + sub-Airy	Annular (ε = 0.5)	0.7

Supplementary Table 3. Subject information

Subject ID	Sex	Eye imaged*	Axial length (mm)	Refractive error, spherical equivalent (D)
Subject 1	F	OD	22.60	-0.375
Subject 2	M	OD	24.66	-0.50
Subject 3	M	OS	23.64	+0.375

*OD – right eye, OS – left eye
