# Supplementary material for: Surpassing the diffraction limit for improved lateral resolution in adaptive optics optical coherence tomography of the living human eye
Source: Commun Eng. 2025 Dec 29;5:3. doi: 10.1038/s44172-025-00573-5 (PMC12764984; doi:10.1038/s44172-025-00573-5)
Supplement: Supplementary file 2 — Description of Additional Supplementary Files [file 44172_2025_573_MOESM2_ESM.pdf]

Description of Additional Supplementary Files:

File: Supplementary Movie 1

Description:

3D AOOCT volumes of the foveal cone mosaic compared under different resolution-enhancement conditions. Left Panel: B-scan (cross-section) view of the fovea (Subject 1). Right panel: maximum intensity projection of the inner segment/outer segment junction (IS/OS) photoreceptor layer showing the en face view of the foveal cone mosaic. Under the conventional condition (full pupil illumination and conventional detection), photoreceptors are increasingly difficult to delineate near the foveal center and a characteristic speckle pattern is observed. With the introduction of a sub-Airy pinhole (0.7 ADD), punctate reflections in OCT B-scans (left panel) are observed in the IS/OS photoreceptor layer and individual photoreceptors can be resolved across much of the field in en face images projected through the IS/OS layer (right panel). Further addition of an annular pupil ($\varepsilon = 0.5$) in the illumination path (ring + sub-Airy condition) leads to further improvement, where cones closer to the foveal center with the smallest cell spacing can be better resolved compared to the conventional or sub-Airy conditions.
