# Supplementary material for: Surpassing the diffraction limit for improved lateral resolution in adaptive optics optical coherence tomography of the living human eye
Source: Commun Eng. 2025 Dec 29;5:3. doi: 10.1038/s44172-025-00573-5 (PMC12764984; doi:10.1038/s44172-025-00573-5)
Supplement: Supplementary file 4 — Reporting Summary [file 44172_2025_573_MOESM4_ESM.pdf]

Corresponding author(s): Johnny Tam
Last updated by author(s): 2025/9/30

Reporting Summary

Nature Portfolio wishes to improve the reproducibility of the work that we publish. This form provides structure for consistency and transparency in reporting. For further information on Nature Portfolio policies, see our Editorial Policies and the Editorial Policy Checklist.

Statistics

For all statistical analyses, confirm that the following items are present in the figure legend, table legend, main text, or Methods section.

n/a	Confirmed	
☐	☒	The exact sample size (n) for each experimental group/condition, given as a discrete number and unit of measurement
☒	☐	A statement on whether measurements were taken from distinct samples or whether the same sample was measured repeatedly
☒	☐	The statistical test(s) used AND whether they are one- or two-sided<br>*Only common tests should be described solely by name; describe more complex techniques in the Methods section.*
☒	☐	A description of all covariates tested
☒	☐	A description of any assumptions or corrections, such as tests of normality and adjustment for multiple comparisons
☒	☐	A full description of the statistical parameters including central tendency (e.g. means) or other basic estimates (e.g. regression coefficient) AND variation (e.g. standard deviation) or associated estimates of uncertainty (e.g. confidence intervals)
☒	☐	For null hypothesis testing, the test statistic (e.g. F, t, r) with confidence intervals, effect sizes, degrees of freedom and P value noted<br>*Give P values as exact values whenever suitable.*
☒	☐	For Bayesian analysis, information on the choice of priors and Markov chain Monte Carlo settings
☒	☐	For hierarchical and complex designs, identification of the appropriate level for tests and full reporting of outcomes
☒	☐	Estimates of effect sizes (e.g. Cohen's d, Pearson's r), indicating how they were calculated

Our web collection on statistics for biologists contains articles on many of the points above.

Software and code

Policy information about availability of computer code

Data collection	AO data collection was performed using custom software as described in the manuscript (Methods).
Data analysis	Adobe photoshop, Matlab, and custom-written software for data visualization and image analysis as described in the methods section.

For manuscripts utilizing custom algorithms or software that are central to the research but not yet described in published literature, software must be made available to editors and reviewers. We strongly encourage code deposition in a community repository (e.g. GitHub). See the Nature Portfolio guidelines for submitting code & software for further information.

Data

Policy information about availability of data

All manuscripts must include a data availability statement. This statement should provide the following information, where applicable:

- Accession codes, unique identifiers, or web links for publicly available datasets
- A description of any restrictions on data availability
- For clinical datasets or third party data, please ensure that the statement adheres to our policy

Data supporting the findings of this study are available within the paper and its supplementary information files.

Research involving human participants, their data, or biological material

Policy information about studies with human participants or human data. See also policy information about sex, gender (identity/presentation), and sexual orientation and race, ethnicity and racism.

Reporting on sex and gender	The study included 2 males and 1 female (Supplementary Table 3).
Reporting on race, ethnicity, or other socially relevant groupings	The study includes 1 Caucasian, 1 Asian, and 1 participant of multiple races.
Population characteristics	The participants of the study included subjects with no signs of ocular diseases.
Recruitment	All subjects were recruited from the National Eye Institute Eye Clinic at the National Institutes of Health, and written informed consents were obtained prior to imaging.
Ethics oversight	The study was approved by the institutional review board of the National Institutes of Health and was conducted in accordance with the Declaration of Helsinki.

Note that full information on the approval of the study protocol must also be provided in the manuscript.

Field-specific reporting

Please select the one below that is the best fit for your research. If you are not sure, read the appropriate sections before making your selection.

☒ Life sciences ☐ Behavioural & social sciences ☐ Ecological, evolutionary & environmental sciences

For a reference copy of the document with all sections, see nature.com/documents/nr-reporting-summary-flat.pdf

Life sciences study design

All studies must disclose on these points even when the disclosure is negative.

Sample size	This study uses new imaging technologies to assess imaging resolution improvement within 3 eyes of 3 subjects.
Data exclusions	Since adaptive optics video sequences are captured, we excluded any raw data captured during blinks, excessive eye motion, or other drops in image quality that prevented image sequences from being registered to a reference volume which is the standard approach for processing adaptive optics retinal imaging data.
Replication	The proposed imaging techniques were applied to a cohort of healthy subjects, demonstrating the repeatability of the approach. AO-OCT volumes were successfully acquired from all subjects included in the study at multiple retinal eccentricities.
Randomization	Randomization was not applicable as all imaging conditions were tested in each eye imaged for the study, allowing within subject analysis.
Blinding	Blinding was not applicable as all imaging conditions were tested in each eye imaged for the study, allowing within subject analysis.

Reporting for specific materials, systems and methods

We require information from authors about some types of materials, experimental systems and methods used in many studies. Here, indicate whether each material, system or method listed is relevant to your study. If you are not sure if a list item applies to your research, read the appropriate section before selecting a response.

Materials & experimental systems

n/a	Involved in the study
☒	☐ Antibodies
☒	☐ Eukaryotic cell lines
☒	☐ Palaeontology and archaeology
☒	☐ Animals and other organisms
☒	☐ Clinical data
☒	☐ Dual use research of concern
☒	☐ Plants

Methods

n/a	Involved in the study
☒	☐ ChIP-seq
☒	☐ Flow cytometry
☒	☐ MRI-based neuroimaging

Plants

Seed stocks	N/A
Novel plant genotypes	N/A
Authentication	N/A
